# Structural diversity and technological trajectories in the agrarian sector in the Amazon: ideas and contexts. A testimony by Francisco de Assis Costa

**DOI:** 10.1590/0102-311XEN229623

**Published:** 2025-03-24

**Authors:** Antonio Miguel Vieira Monteiro, Danilo Araújo Fernandes, Cláudia Torres Codeço, Francisco de Assis Costa

**Affiliations:** 1 Instituto Nacional de Pesquisas Espaciais, São José dos Campos, Brasil.; 2 Universidade Federal do Pará, Belém, Brasil.; 1 Programa de Computação Científica, Fundação Oswaldo Cruz, Rio de Janeiro, Brasil.

**Figure:**
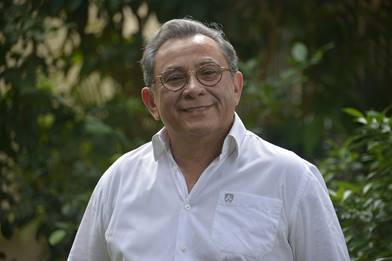
Professor Francisco de Assis Costa (photo: Irene Almeida).

This testimony, which makes up the Supplement *Synthesis Trajectories: Relations Between Health and Environment in the Contemporary Amazon*, was born from an interview with Chiquito, as we affectionately know Professor Francisco de Assis Costa, about the concept of “techno-productive rural trajectories”, its origin, and its applications for the study of the relations between man and the environment in the Amazon. Economist, professor, and researcher at the Center for Higher Amazonian Studies (Naea, acronym in Portuguese) at Federal University of Pará (UFPA, acronym in Portuguese), Chiquito has dedicated his career to studying the economic history of the Amazon, acknowledging farm laborers as the economic agents of a strong and resilient economy. Farm laborers and their economy have been present in the biome for at least 250 years, but made invisible due to the various dimensions of macroeconomic policies and development strategies in dispute for the region. His theory and worldview enrich the thought of determining health in the Amazon by finding different forms of relationship between humanity and the environment, which are expressed in their ways of existing and producing in the Amazon biome.

We asked Chiquito some guiding questions: “Chiquito, before we move on to the topic, can you briefly describe your academic trajectory and your current work?”; “Can you explain the concept of technoproductive trajectory and why it is important to give visibility to the structural diversity of life in the Amazon?”; “The Forest is seen by many as a source of poverty and disease. It is common to use low income or Human Development Index (HDI) as indicators of poverty in models of social determination of health. How do you reflect on the use of these indicators in the context of Amazonian structural diversity?”; “Chiquito, at the center of your theory is the category of the ‘Amazonian caboclo farm labor’. In what technoproductive trajectories are they found and how do technoproductive trajectories affect the survival and quality of life of this population?”. Chiquito was very motivated and enthusiastic about the proposed questions. The reason, he told us, was that the questions made a lot of sense for him as a set, rather than in isolation. They led him to recover the historical path of construction of his categories and his understanding of the agrarian economy of the contemporary Amazon. Chiquito then proposed that instead of answering the questions, one by one, he could write a text gathering the questions in the form of a personal testimony about the birth and evolution of an idea. Chiquito thus offers us, with this interview-testimony, the history of the development of his thought, which is embedded and inseparable from his historical time.

Danilo: Can you explain the concept of techno-productive trajectory and why it is important to give visibility to the structural diversity of life in the Amazon? In your answer, can you tell us how this theme enters into your academic trajectory?

Chiquito: The concurrent trajectories-based approach to development adopted in our research on the Amazon is a way of addressing structural diversity in Brazilian rural formation, a seminal theme in the Brazilian social sciences. The processes of this elaboration involved multiple actors and contexts in a long path of social construction.

The discussion on structural diversity in rural areas and its meanings in Brazilian development had a particularly important moment in the transition from the 1970s to the 1980s, in a particularly flourishing context and moment in the social sciences of the country. History, anthropology, sociology, economics, geography, all sought in some way to respond to the interpretative demands of a singularly tense reality in the economic sphere − due to the growth model that, for more than a decade, gave rise to new levels of industrialization in the country and unprecedented forms of rural reconfiguration associated with it − and in the political sphere − as the experienced structural dynamics depended on an authoritarian dictatorial state. Whether in its expansion and boom phase (the so-called Brazilian miracle occurred from 1968 to 1973), or in its deceleration phase (from 1974 to 1979) and crisis (established in 1982), the economic model had a tremendous impact on the Brazilian social structure, profoundly transformed its agriculture, and reformatted the conditions by which its most dynamic regions related to the peripheral ones.

At the same time, the Brazilian social sciences saw their staff expand with the universities, which grew in the same period in number, size, thematic scope, and territorial coverage. Part of the growth of higher education was explained by the needs of agricultural development, whether in terms of agronomists, mechanics and chemists to ensure technical advances, or in terms of economists, anthropologists, sociologists, and public administrators to understand, plan, monitor, and control the sector. On the other hand, the contradictions of this same growth process, creating unstable labor relations in the countryside, deepening land concentration, and expanding the masses of displaced people from the land challenged the growing capacity, should be reaffirmed for academic reflection. A phenomenon that emerges from the confluence between the growth of the State needs, the increase in the Brazilian university capacity, and the broadening and further development of reflection refers to the Agricultural Development Graduate Center (CDPA, acronym in Portuguese).

Installed in 1976, with funding from the Brazilian Ministry of Agriculture, the CDPA was created to train high-level staff for the National Agricultural Planning System (SNPA, acronym in Portuguese), under the coordination of the National Secretariat for Agricultural Planning (SUPLAN, acronym in Portuguese) ^1^. Since 1975, the SUPLAN had sponsored specialization courses in agricultural planning for agronomists, economists, sociologists, and administrators to train technical staff for the State Commissions for Agricultural Planning (CEPA, acronym in Portuguese) and the Regional Coordination for Agricultural Planning (CRPA acronym in Portuguese). I participated in the first specialization course in Agricultural Planning of this extensive program, held in Belém (Pará State) from May 12 to September 12, 1975 ^2^. The initiative had the support of the Food and Agricultural Organization of the United Nations (FAO), the Economic Commission for Latin America and the Caribbean (ECLAC), the Superintendence for the Development of the Amazon (SUDAM, acronym in Portuguese) and the Superintendence for the Development of Brazilian Northeast (SUDENE, acronym in Portuguese). The CDPA was envisioned as a higher stage in this effort, with a master’s degree teaching component and a research apparatus on the major issues surrounding agriculture ^1^.

The CDPA was installed at the Inter-American School of Public Administration at Getúlio Vargas Foundation (FGV, acronym in Portuguese) in Rio de Janeiro. In the same year that he set up his Master’s Degree in Agricultural Development (CMDA, acronym in Portuguese), in 1977, two research programs were set in motion: the History of Agriculture Program and the Project for the Recent Evolution and Current Situation of Brazilian Agriculture (Persagri, acronym in Portuguese).

In a first phase, the CDPA curriculum was strongly influenced by the FGV Graduate School of Economics (EPGE, acronym in Portuguese). Its strongly disciplinary tradition with a radically neoclassical bias, one of the first higher education units in the country with graduate education, which has offered master’s economics courses since 1966 and PhD courses since 1974, created growing tensions with the body of students from varied backgrounds and, for the most part, experienced agronomists from technological development and technical assistance institutions or economists specializing in planning with structuralist training in the ECLAC tradition, placed at the disposal of the National Agricultural Planning System at its different levels. No less important tensions were formed between this academic teaching proposal and the staff of researchers who were primarily hired for History of Agriculture Program and Persagri, mostly coming from history, anthropology, political economy, and other heterodox lines of economics.

The worsening of tensions led, as early as 1978, to important changes in the curriculum, resulting in a reduction of the spaces of neoclassical micro and macroeconomics, in the recognition of Keynesian economics to understand development and the developing State, and in the progressive increase in the space of contributions from Marxists and the ECLAC. In this new context, economic approaches will be continuously strained by the agrarian question addressed by historians, anthropologists, political scientists, and sociologists ^1^.

This state of affairs was reflected in the profile of the institution’s teaching staff. The CDPA constituted a critical mass of unique characteristics in the academic history of Brazil, with a high level of training that was diversified both in terms of discipline and doctrinal and thematic orientation, including economists from a variety of backgrounds - from those with a neoclassical orientation (such as Affonso Celso Pastore - a macroeconomist - and Antônio Carlos Nogueira - a rural economist,) to those with a diversified heterodox orientation (such as Carlos Lessa, Yoshiaki Nakano, José Pereira Wilken Bicudo, Paulo Roberto Beskow, Nelson Giordano Delgado, Ana Célia Castro, and Ivan de Otero Ribeiro); sociologist Miriam Limoeiro Cardoso, agronomists Horácio Martins de Carvalho and Roberto Moreira (both with a strong interdisciplinary background, the former a specialist in planning with recognized capacity in political science, the latter with a solid background in political economy); anthropologists Otavio Guilherme Velho and Margarida Maria Moura; and historians Maria Yedda Leite Linhares, Guillermo Palacios, and Francisco Carlos Teixeira da Silva. It is worth mentioning that this body of professors and researchers, in pairs, sometimes multiple institutional affiliations, articulated the CDPA to different centers of thought; for example, Affonso Celso Pastores, a professor of economics at São Paulo University (USP, acronym in Portuguese); Carlos Lessa, in turn, professor at Economic Commission for Latin America and the Caribbean and at Federal University of Rio de Janeiro (UFRJ, acronym in Portuguese); Yoshiaki Nakano, from the School of Economics at FGV in São Paulo; Otávio Guilherme Velho, at the time a prominent professor of anthropology at the National Museum of UFRJ; and Miriam Limoeiro Cardoso, an important professor of sociology at Pontifical Catholic University (PUC, acronym in Portuguese) of Rio de Janeiro, etc.

This phase, which goes from its creation until 1981, when it begins a process of transfer to Rural Federal University of Rio de Janeiro (UFRRJ, acronym in Portuguese), is known as “Horto CDPA,” in allusion to the fact that it operates at Horto Florestal, in the manor house of the former residence of the monarchy in Rio de Janeiro, currently Solar da Imperatriz ^1^. The Horto CDPA criticized the earlier dominant approaches to agriculture in Brazil, contributing to the broad debate that was beginning in academia about the singularities of the Brazilian development, especially in its agrarian dimension. At the same time, it proposed paths for a new interpretation of this object. The aforementioned research programs were efficient catalysts for this movement.

The History of Agriculture Program was directed by Professor Maria Yedda Leite Linhares. Professor at the former University of Brazil, now UFRJ, compulsorily retired by the military dictatorship in 1969, associate professor at Paris-Vincene and at the University of Toulouse-Le Mirail in France. Since then, up to her return to Brazil in 1974, Professor Maria Yedda pioneered the area of the history of agriculture in our country, creating research programs, as mentioned, at the Fundação Getulio Vargas Intramerican School of Public Administration, and then, after her full reinstatement as a professor of federal public teaching at Fluminense Federal University (UFF, acronym in Portuguese) and at UFRJ. In her fruitful career, she supervised dozens of dissertations and theses, including my master’s thesis.

Professor Maria Yedda gathered many collaborators and the CPDA staff around the History of Agriculture Program, including Alfredo Wagner Berno de Almeida, Ciro Flamarion S. Cardoso, Maria Bárbara Levy, Eulália Maria Lahmeyer Lobo, Fernando Antônio Novais, Francisco José Calazans Falcon, and João Pacheco de Oliveira Filho ^1^. Moreover, the History of Agriculture Program held a weekly event of discussions in which permanent or partial local professors and researchers and scholars such as Waren Dean, Otavio Ianni, José Manoel Cardoso de Melo, Manoel Maurício, Wilson Cano, Afrânio Garcia, Klaas Wortman, Shepard Forman, Ismênia Martins, Ilmar Roloff Mattos, Alice Canabrava, and Maria Rita Garcia Loureiro paraded their works in the halls of the former monarchy in the Horto Florestal of Rio de Janeiro. About the vibrant atmosphere of those days, in 2007, professor Maria Yedda recalled: “*Young people in search of knowledge, ideas, or reaffirmation made up a permanent picture in the gardens of the mansion. Witold Kula, Georges Duby, Fernand Braudel, Karl Polany, and Maurice Godelier were eagerly read and discussed regarding the direction of the country on the eve of the collapse of the dictatorship*” ^1^ (p. 166).

In the History of Agriculture Program at the Horto CPDA, it had become urgent to confront the theoretical and methodological restrictions that characterized agrarian historiography in Brazil, especially the limits imposed by the dualistic interpretations of the structural diversity of the Brazilian reality developed in recent decades.

A first line of questioning referred to the approach that favored export agriculture, obscuring everything else. Since the seminal work of Roberto C. Simonsen, in 1937 ^3^, which divided the colonial period into cycles dominated by a product or activity - the “extractive industry cycle”, with emphasis on “brazilwood”; the “sugar cycle,” and the “mining cycle” - the economic history of the country was approached by successions of these cycles: the “coffee cycle”, the “rubber cycle”, etc. Such a perspective compromised the historical view of the country, whether in terms of its structural foundations or in terms of its movements and evolution over time.

In structural terms, the centrality or even exclusivity of the export economy (brazilwood, sugar, mining, coffee) implied suppressing the production for domestic supply (rice, corn, beans, cassava, cachaça) and, with this absence, the effective presence of structures other than the plantation immediately responsible for the cycle. A particularly noticeable absence was that of the structures of small family producers since evidence of their importance was gathered (with varying weights) in practically all parts and moments of the history of the country. This was reported by the works by Ciro Flamarion Cardoso on the farm laborer gap in slave-owning Brazil, by Antônio Cândido on the caipiras of São Paulo, by Margarida Maria Moura, on the farm laborers of Minas Gerais, by José de Souza Martins on the farm laborers on the northern border of Paraná, and by Otávio Guilherme Velho, João Pacheco de Oliveira, and Alfredo Wagner Berno de Almeida on farm laborers in the Amazon ^4^ ^,^ ^5^ ^,^ ^6^ ^,^ ^7^.

Furthermore, as an orientation of the temporal movement of social reality, the notion of cycle carried with it a teleological content, whereby the highlighted product necessarily showed a complete life cycle - it arises, evolves, decays, and disappears. However, this was not the case for any of the important products for the economic history of the country - except for the noble exception of brazilwood.

A second line of limiting issues for the history of agriculture in Brazil, according to the reading of “Horto CDPA”, concerned the dualism of agriculture in Brazilian society as a whole - another perspective of structural diversity. Initially popularized by Jacques Lambert and Roger Bastide, professors at the School of Sociology and Politics at USP, it stated that countries with a colonial past would show a duality of structures, with an “open and modern” sector and a “closed and archaic” one. In Brazil, therefore, the old, represented by the immobility of the interior, the archaic rural inherited from the colonial past as the survival of outdated forms, and the new, represented by the dynamism of the modern coast, with its urban structures that were linked to industry, large commerce, and advanced forms of civilization, would be opposed. The explanation for this reality would be in the colonization that generated the latifundium of feudal character, rigidly hierarchical, constituting self-sufficient units, whose predominant trait would be resistance to change ^8^ ^,^ ^9^.

At Higher Institute of Brazilian Studies [Instituto Superior de Estudos Brasileiro], Hélio Jaguaribe, Nelson Werneck Sodré, Guerreiro Ramos, Ignácio Rangel, among others, produced a Marxist absorption of these theses, greatly influenced by the official perspective of Stalinism. According to this version, the historical trajectory of Western societies would include a rigid succession of modes of production. Applied to Brazil, the notion led to the perception that its economic and social structures would be characterized by the coexistence of two modes of production - the capitalist (modern, manifest in industry and other urban sectors) and the feudal (archaic, established in latifundium agriculture and mini-landowner forms - i.e., large establishments based on non-capitalist lease relations, settlements or partnership, and small farm laborer establishments). From the perspective that, in this context, came to be established as a “national-developmentalist” approach, the national capitalist sector (and the leadership of its leaders, a nationalist bourgeoisie) would depend on the development of the country, in opposition to foreign capitalist companies (an expression of imperialism) and landowning fiefdoms. This process of development would include an agrarian reform with a double purpose: to guarantee the supply of the domestic food market at low prices. For this purpose, it was necessary to expand farm labor and increase its productivity; and, with the increase in the purchasing power of the farm laborers, to increase the domestic market for industrial goods from the national industry ^10^ ^,^ ^11^ ^,^ ^12^.

In the decade that followed the military coup of 1964, the dualist schemes of interpretation of Brazil proved to be fragile, not least because their theoretical assumptions - the feudal character of rural structures and nationalism as an instrument for conducting policy against imperialism - were not empirically proven. Furthermore, the development model that followed in the coming years, based on industry, on an arrangement that proved virtuous in promoting economic growth among foreign companies, State-owned companies, and national sectors, and, in agriculture, on the technological modernization of the large latifundia and related changes in the relations of production, showed the impropriety of that analytical scheme.

Two lines of critical confrontation initially reached dualism. One supported by methodological individualism instrumented by neoclassical approaches to the development of the country and agriculture and the other structuralist, with a Marxist foundation.

The neoclassical perspective, partially formulated at USP under the leadership of Antônio Delfim Netto, Affonso Celso Pastore, among others, and at the Brazilian Institute of Economics at FGV, by Rui Miller Paiva, the Brazilian rural sector would be populated by agents whose decisions would be compatible with orthodox economic theory. Therefore, the dualist assumptions of obstacles derived from non-capitalist reasons and modes of production would be irrelevant. So much so that the sector, instead of constituting an obstacle, was, as a whole, reacting to the tensions that urban and industrial growth exerted on it, according to the theoretical expectations of market analyses: it had been raising productivity and, thus, producing food at adequate prices and freeing up labor for industry. The sector also provided resources for the formation of capital in the industry and constituted a base for the expansion of the consumer market. Finally, the different endowments and productivity of factors would explain the verifiable differences between regions and within them, between activities ^13^ ^,^ ^14^ ^,^ ^15^ ^,^ ^16^.

The Marxist front of criticism of dualism had its vanguard in two authors. Caio Prado Júnior ^17^, professor of political economy at USP, who, in the early 1940s, argued that the origins of Brazil were capitalist since the Portuguese colonial enterprise was part of the context of the expansion of mercantile capitalism, resumes in 1966 the theses defended at the time and updates them to a context in which industrial capitalism advances in the country under the leadership of foreign companies, with unavoidable consequences in agriculture ^18^ ^,^ ^19^. André Gunder Frank, in an article published a little earlier, in 1964, also starting from the capitalist character of colonization, argues that the industrialization of Brazil evolves subordinated to imperialism and with severe technological dependence under the aegis of capitalism, which organizes the whole of society, including its agriculture. From this perspective, it is the functioning and advancement of this system that necessarily produces both development and underdevelopment.

All these discussions strongly reverberated from 1977 to 1981 at Horto CDPA, and they formed the basis for reflections in many directions. First, it bothered me that, on both fronts, with the rejection of what was criticizable in dualism - a fundamental diversity understood as distinct realities side by side, watertight in relation to each other - all difference was annulled, to the extent that a status of necessary transience was established for the diverse: in the end, if diversities existed, they were irrelevant remnants of anachronistic forms that insisted on continuing to exist, but not for long. Notwithstanding the explanatory differences (whether diversity derives from the existence of rational and non-rational agents or from archaic structures in coexistence with modern ones), everyone tended to see the distinct as necessarily (logic or history would be implacable) and desirably (the best becoming presupposed its extinction) transitory. So quickly would surpassing instances occur, everyone believed, neoclassical, Marxist, developmentalist, that we should proceed as if they, the distinct, no longer mattered.

At Horto CDPA, the criticism of these thoughts gave rise to a re-discussion of Marxism and its fundamental notions of modes of production and economic-social formations, even starting from the criticism of structuralism due to the growing use of the so-called Chapter VI of *O Capital*, a manuscript by Marx, unpublished until the beginning of the 1960s and published in Brazil in 1978 ^20^. It is important to point out that this rereading considered the theoretical and methodological contributions of the Annales School, bases of the very important course in the history of agriculture taught by Professor Maria Yedda Linhares. From there, the question was asked about the capacity of the capitalist development process to functionalize, create, and recreate pre-existing or emerging non-capitalist forms of production and, thus, contain in its modes of evolution long-term structural diversities.

From this emerge ontologies that will inquire about Brazilian agriculture as a multifaceted reality, which must be understood in the specificity of the economic and social formation of the country, which, although under the hegemony of capitalism, articulates different relations and modes of production. In more detail, these big hypotheses would be ^21^:

1) Brazilian agriculture was constituted in the context of the capitalist development of the country characterized as dependent and peripheral. The backward technical forms and the multiplicity of labor relations in the countryside would not be explained as colonial survivals, but rather as forms of productive organization functionalized by the very rationality of dependent and peripheral capitalism; 

2) Capitalist forms of production were increasingly asserting themselves on the basis of different forms of direct subordination of labor, from stable wage labor in certain circumstances and functions to temporary mobile labor that tended to become generalized in the great culture of export; 

3) Forms of production in farm labor take different formats and contexts: producing food as small parcel farmers in the most depressed areas of old colonization as small rural entrepreneurs in more dynamic old areas, or even as squatters in new areas of the country, on their agricultural frontier.

The Amazon will be problematized in this discussion, in the late 1970s and early 1980s, as the most recent agricultural frontier area in the country. As such, it will be characterized, as the works of Otávio Guilherme Velho and José de Souza Martins will show, as other agricultural frontiers by farm laborer frontiers of diverse characteristics and origins in processes of spontaneous or directed agricultural colonization ^22^ ^,^ ^23^ ^,^ ^24^. However, Roberto de Araújo Santos, João Pacheco de Oliveira Filho, and Armando Dias Mendes will soon show that local caboclo populations, grossly disregarded in the Brazilian discussion, composed structures prior to this frontier movement, bringing to the process their own characteristics ^25^ ^,^ ^26^ ^,^ ^27^.

The Amazonian frontier will also be distinct in this phase, as indicated by Otávio Ianni, Lúcio Flávio Pinto, and José de Souza Martins, due to the growing participation of capitalist agricultural enterprises supported by state incentive mechanisms for land, tax, and credit concessions ^28^ ^,^ ^29^ ^,^ ^30^ ^,^ ^31^. The capitalist component of the Amazonian frontier was an unprecedented phenomenon in the rural formation of the country that laid outside the analytical schemes that follow the modernization of latifundia because it was not the result of processes of evolution of pre-existing rural structures - it was not the initiative of local farmers, cattle ranchers from Marajó, for example, who promoted modernization, or coffee farmers from São Paulo, or sugarcane from Pernambuco, or cocoa from Bahia, in processes of expansion or change in the distant Amazon, but urban companies from the main industrial and service centers of the country in a stimulated process of ruralization.

This form of evolution of the Amazonian frontier, in its decisive elements, had begun at the end of the 1960s, with the consolidation of the set of policies the military dictatorship had imposed on the region. This Operation Amazon [*Operação Amazônia*], as it was called at the time, included land, tax, and credit instruments coordinated by the SUDAM, which favored large industrial and service companies that would act as agricultural companies in the Amazon. However, at the end of the 1970s, the results of the strategy were unclear since no company had yet consolidated itself. The observation of future prospects was clouded by the strong restriction on access to information from the institutions involved in this particularly sensitive operation of a State that was particularly obscure in its authoritarian ethos. Nevertheless, two master’s dissertations from Horto CDPA investigated the meaning of this unusual presence of large urban capital on the agricultural frontier.

Maria das Graças Derengovsky Fonseca ^32^, under the advise of José Graziano da Silva, undertook a courageous and important field research to try to capture, in direct interviews, the course of things. Her work ended up qualifying the movement as adventurous. My master’s thesis, in the context of History of Agriculture Program and under the guidance of Professor Maria Yedda Linhares, sought to indirectly access the meaning of the phenomenon, rescuing the history of the Ford project in the Amazon ^33^.

Researching the experience of the Ford Motor Company in the Amazon in the 1920s seemed like a way to understand what was going on. It occurred to us, to my advisor, to my co-advisor, Professor Horácio Martins de Carvalho, and to me, that this impressive experience, at that time, very little studied, represented a prototype of the experiments now underway in the region under the baton of SUDAM: in essence, then as now, they were capitalist enterprises based on large plantations or extensions of pasture dealing, from a strict industrial logic, with the natural and social peculiarities of the region - these, frankly misunderstood, both in the time of Ford, and in the times of authoritarian modernization of the 1970s, the stage of our academic experience.

Once the research was carried out, it was found that our assumptions were correct: Ford’s experience in the 1920s and 1930s was dotted with situations equivalent to phenomena in progress in the 1970s in the SUDAM experience, particularly the social tensions around the work of an extensive and multifaceted farm labor and the technological difficulties that, together, ended up making the great enterprise fail. The final warning the work offered to the protagonists of the project led by SUDAM, half a century later, was blunt: the one in force in the 1970s and 1980s could repeat the failure of Ford, possibly for the same reasons. With these first exercises, I wished to test the hypothesis that a deep structural diversity, which harbors interactions between modes of production, with events of negation, recreation, and historical emergence - thus without room for teleologies, as per Horto CDPA - configure a long-term characteristic of the Brazilian rural formation that manifested itself strongly in its Amazonian moment. This arrangement has organized my work program ever since, starting with my doctoral thesis.

Danilo: At the center of his theory is the category of the “Amazonian caboclo farm labor”. At what point is this centrality established? In which technoproductive trajectories are they found and how do technoproductive trajectories affect the survival and quality of life of this population?

Chiquito: I have so far described the background of my research since my PhD. There, at the economics department of the Freie Universität Berlin, from 1984 to 1988, I investigated the formation of Amazonian farm labor from the colonial period to the 1980s. Back in Brazil, I developed studies on the experience of SUDAM, on the ruralization in the Amazon of large industrial, banking, and commercial companies, confronting this experience, which, in fact, proved to be a failure, with the emergence of new and different economically viable employer or capitalist structures. Which were characterized by being technologically backward; I also analyzed the evolution of different forms of farm labor, which, on the contrary, reemerged from crises on different frontiers due to unexpected movements of technological innovation of great creativity, extension, and scope ^34^.

Part of this effort consisted of developing theoretical and methodological capacity to treat structural diversity as a result of the structure-reason-action interaction, initially accepting the suggestion of Antonio Carlos Kfouri Aidar & Roberto Mário Perosa Junior ^35^ on the relevant differences in the decision-making reasons of profit-driven agents given that they can follow a more mercantile, or more industrial, or more financial logic, implying, therefore, the different technological results that we saw between the types of companies. I soon could understand that these distinctions would be treatable from the perspective of John Maynard Keynes ^36^, of the search for marginal efficiency of capital by the composition of variable portfolios, contingent in time and space. On the other hand, I developed, based on the seminal work of the Russian economist Alexeder von Chayanov on the economic rationality of the domestic economy, the category of reproductive efficiency, from which it was possible to explain the surprising and varied technological dynamics and results we found in farm laborers ^37^.

With this, I acquired the ability to visualize the rural formation of the Amazon, on the one hand, as a result of the evolution of forms of farm laborer, that can be gathered into a farm laborer mode, in interaction with employer forms, that can be gathered into an employer mode of production organization; on the other hand, as a process in which these modes of production observable at a high level of abstraction showed a great diversity of movements, structural situations, and concrete technological solutions. Our studies up to 2005 on the agricultural formation of the Brazilian North and floodplains represent what was achieved with this ^38^ while containing important deficiencies: that of establishing systemic meanings for the variety of ad hoc phenomena that were concretely described and to expose their coherence with their great structural references. 

When I went to the Center for Brazilian Studies at the Saint Antony’s College at the University of Oxford, in 2007, I carried these problems in my luggage. To overcome them in the time I was there, I delved into the works of Paul David ^39^, a prominent man at the institution, on the centrality of technical relations in economic growth and trajectory dependence in the evolution of technological procedures (his central message: history matters), by Giovani Dosi ^40^, on technological trajectories as concrete operations referred to technological or paradigmatic standards (the modes and sources of generation, selection, diffusion and appropriation of technological knowledge matter) and those of Brian Arthur ^41^ on the competition of technological trajectories as a historical process that depends on the institutional environment and the postural differences of the agents (the reasons and modes of production matter). To this set of illuminating results, I added the idea that, to the historicity of knowledge and the institutions they highlight, territorialities are linked and, with them, necessary natural bases (so that place and nature matter) ^29^.

With this, since then, I have had as a working hypothesis that the forms of use of natural and institutional resources, their movements, and conformations we concretely saw and described should be understood as points, moments, and situations of technological trajectories, which could eventually be observed by convergences in time and similarities in the space of these points ^42^. To use the databases of the agricultural censuses and thus be able to delimit these trajectories at different levels of aggregation, we developed a method that combines the differentiation and structural significance of rural production in two movements: in the former, the modes of production are segregated by characteristics of the agents and the situation of their structures and, in the latter, it is verified how these dissimilar structures harbor convergences in the characteristics of production semantically associated with trajectories ^43^.

The results, which already enabled steps in the interdisciplinarity of the social sciences with the successful experience of the Trajectories project: *Ecosystem Services as Health Services: Competitive Trajectories for Land use in the Amazon Biome and its Link to Vector-borne Diseases*, promoted by the Brazilian Center for Synthesis on Biodiversity and Ecosystem Services by Brazilian National Research Council (SinBiose/CNPq, acronym in Portuguese), seems to guarantee leaps in the transdisciplinarity necessary for the complex treatment the development of the Amazon, this critical region of the planet, deserves.
