# Supplementary material for: Structural diversity and technological trajectories in the agrarian sector in the Amazon: ideas and contexts. A testimony by Francisco de Assis Costa
Source: Cad Saude Publica. 2025 Mar 24;41(Suppl 1):e00229623. doi: 10.1590/0102-311XEN229623 (PMC11940857; doi:10.1590/0102-311XEN229623)
Supplement: Translation

# Translation: Diversidade estrutural e trajetórias tecnológicas no agrário da Amazônia: ideias e contextos. Depoimento de Francisco de Assis Costa

**DOI:** 10.1590/0102-311XPT229623

**Authors:** Antonio Miguel Vieira Monteiro, Danilo Araújo Fernandes, Cláudia Torres Codeço, Francisco de Assis Costa

**Affiliations:** 1 Instituto Nacional de Pesquisas Espaciais, São José dos Campos, Brasil.; 2 Universidade Federal do Pará, Belém, Brasil.; 3 Programa de Computação Científica, Fundação Oswaldo Cruz, Rio de Janeiro, Brasil.

**Figure:** Professor Francisco de Assis Costa (foto: Irene Almeida).

Este depoimento, que compõe o Suplemento *Trajetórias de Síntese: Relações entre Saúde e Ambiente na Amazônia Contemporânea*, nasceu de uma entrevista com o Chiquito, modo como carinhosamente conhecemos o professor Francisco de Assis Costa, sobre o conceito de “trajetórias tecnoprodutivas rurais”, sua origem e suas aplicações para o estudo das relações do homem com o meio ambiente na Amazônia. Economista, professor e pesquisador do Núcleo de Altos Estudos Amazônicos (Naea) da Universidade Federal do Pará (UFPA), Chiquito dedicou sua carreira ao estudo da história econômica da Amazônia, reconhecendo os camponeses como os agentes econômicos de uma economia forte e resiliente. Presentes há, pelo menos, 250 anos no bioma, mas invisibilizados, os camponeses e sua economia, pelas várias dimensões das políticas macroeconômicas e estratégias de desenvolvimento em disputa para a região. Sua teoria e visão de mundo enriquecem o pensamento da determinação da saúde na Amazônia, a partir da identificação de diferentes formas de relação do homem com o ambiente, expressas em seus modos de existir e produzir no bioma Amazônico.

Formulamos ao Chiquito algumas perguntas norteadoras: “Chiquito, antes de partirmos para o tema, pode descrever brevemente sua trajetória acadêmica e sua atuação atual?”; “Pode nos explicar o conceito de trajetória tecnoprodutiva e por que ele é importante para dar visibilidade à diversidade estrutural da vida na Amazônia?”; “A Floresta é vista por muitos como fonte de pobreza e doença. É comum usar baixa renda ou Índice de Desenvolvimento Humano (IDH) como indicadores de pobreza em modelos de determinação social da saúde. Qual é a sua reflexão sobre o uso desses indicadores no contexto da diversidade estrutural Amazônica?”; “Chiquito, no centro de sua teoria está a categoria do ‘campesinato caboclo Amazônico’. Em que trajetória tecnoprodutiva é encontrada e como elas afetam a sobrevivência e qualidade de vida dessa população?”. Chiquito ficou muito motivado e entusiasmado com as questões propostas. A razão, nos disse, era que as perguntas, para ele, em conjunto, faziam muito sentido, mas não isoladamente. Elas o levaram a recuperar o percurso histórico de construção de suas categorias e sua compreensão sobre a economia agrária da Amazônia contemporânea. O professor nos propôs, então, se em vez de responder as perguntas, uma a uma, ele poderia escrever um texto aglutinando as perguntas, na forma de um depoimento pessoal sobre o percurso de nascimento e evolução de uma ideia. Dessa forma, com essa entrevista-depoimento, Chiquito nos presenteia com a história da formação do seu pensamento, embebido e inseparável de seu tempo histórico.

Danilo: Pode nos explicar o conceito de trajetória tecnoprodutiva e por que ele é importante para dar visibilidade à diversidade estrutural da vida na Amazônia? Em sua resposta, pode nos contar como esse tema entra em sua trajetória acadêmica?

Chiquito: A abordagem do desenvolvimento baseado em trajetórias concorrentes, adotada em nossas pesquisas sobre a Amazônia, é um modo de abordar a diversidade estrutural na formação rural brasileira, um tema seminal das ciências sociais do país. Os processos dessa elaboração envolveram múltiplos atores e contextos em um longo trajeto de construção social.

A discussão sobre a diversidade estrutural no meio rural e seus significados no desenvolvimento brasileiro teve um momento particularmente importante na passagem dos anos setenta para os oitenta, em contexto e momento particularmente florescentes das ciências sociais no país. A história, a antropologia, a sociologia, a economia e a geografia, todas procuravam de algum modo responder às exigências interpretativas de uma realidade singularmente tensa, na esfera econômica, devido ao modelo de crescimento que por mais de uma década ensejou novos patamares de industrialização no país e inéditas formas de reconfiguração rural a isso associada, bem como na esfera política, porque as dinâmicas estruturais experimentadas dependiam de um estado autoritário em momento ditatorial. Seja em sua fase de expansão e auge (o chamado milagre brasileiro ocorrido entre 1968 e 1973), seja na fase de desaceleração (de 1974 a 1979) e crise (que se estabeleceu em 1982), o modelo econômico conduzido impactou tremendamente a estrutura social do país, produziu transformações profundas na sua agricultura e reformatou as condições mediante as quais se relacionavam suas regiões mais dinâmicas com as periféricas.

Ao mesmo tempo, as ciências sociais brasileiras viram seu cenário se expandir com as universidades, que cresceram no mesmo período em número, tamanho, abrangência temática e cobertura territorial. Parte do crescimento do Ensino Superior se explica pelas necessidades do desenvolvimento agrícola, seja em relação ao que era exigido em termos de engenheiros agrônomos, mecânicos e químicos para garantir os avanços técnicos, seja em relação à necessidade da atuação de economistas, antropólogos, sociólogos e administradores públicos para a compreensão, planejamento, acompanhamento e controle do setor. Por outro lado, as contradições desse mesmo processo de crescimento, criando relações de trabalho instáveis no campo, aprofundando a concentração fundiária e ampliando as massas de desalojados da terra, desafiaram a capacidade crescente, reafirma-se, de reflexão acadêmica. Um fenômeno que emerge da confluência entre o crescimento das necessidades do Estado, o aumento da capacidade universitária do país e a ampliação e aprofundamento da reflexão é o Centro de Pós-graduação em Desenvolvimento Agrícola (CPDA).

Instalado em 1976, com financiamento do Ministério da Agricultura, o CPDA foi criado para formar quadros de alto nível para o Sistema Nacional de Planejamento Agrícola (SNPA), sob a coordenação da Secretaria Nacional de Planejamento Agrícola (SUPLAN) ^1^. A SUPLAN vinha, desde 1975, patrocinando cursos de especialização em planejamento agrícola para agrônomos, economistas, sociólogos e administradores, com o objetivo de preparar quadros técnicos para as Comissões Estaduais de Planejamento Agrícola (CEPA) e as Coordenações Regionais de Planejamento Agrícola (CRPA). Participei do primeiro Curso de Especialização em Planejamento Agrícola desse extenso programa, realizado em Belém (Pará), de 12 de maio a 12 de setembro de 1975 ^2^. A iniciativa contava com o apoio da Organização das Nações Unidas para a Alimentação e a Agricultura (FAO), da Comissão Econômica para a América Latina e o Caribe (CEPAL), da Superintendência de Desenvolvimento da Amazônia (SUDAM) e da Superintendência de Desenvolvimento do Nordeste (SUDENE). O CPDA foi pensado como uma etapa superior desse esforço, com um componente de ensino em nível de Mestrado e um aparato de pesquisa sobre as grandes questões que envolviam a agricultura ^1^.

O CPDA foi instalado na Fundação Getúlio Vargas (FGV) do Rio de Janeiro, na sua Escola Interamericana de Administração Pública (EIAP). No mesmo ano em que instalou seu Curso de Mestrado em Desenvolvimento Agrícola (CMDA), em 1977, dois programas de pesquisa foram postos em andamento: o Programa de História da Agricultura e o Projeto de Evolução Recente e Situação Atual da Agricultura Brasileira (Persagri).

Em uma primeira fase, a grade curricular do CPDA teve forte influência da Escola de Pós-Graduação em Economia (EPGE), da mesma FGV-RJ. A tradição fortemente disciplinar com viés radicalmente neoclássico dessa escola, uma das primeiras unidades de Ensino Superior do país com ensino de Pós-graduação, que desde 1966 oferecia cursos de economia em nível de Mestrado e, desde 1974, em nível de Doutorado, criaram tensões crescentes com o corpo de alunos de formações variadas e, em sua maioria, experientes agrônomos das instituições de desenvolvimento tecnológico e de assistência técnica ou economistas especialistas em planejamento com formação estruturalista na tradição da CEPAL, colocados à disposição do SNPA nos seus diferentes níveis. Tensões não menos importantes se formaram entre tal proposta acadêmica de ensino e o quadro de pesquisadores contratados primordialmente para o Programa de História da Agricultura e para o Persagri, na sua maioria provindos da história, da antropologia e da economia política e outras linhas heterodoxas da economia.

O agravamento das tensões levou, já em 1978, a mudanças importantes na grade curricular, resultando numa redução dos espaços das micro e macroeconomias neoclássicas, no reconhecimento da economia keynesiana para a compreensão do desenvolvimento e do estado desenvolvimentista e no aumento progressivo do espaço das contribuições cepalinas e marxistas. No novo contexto, as abordagens econômicas serão continuamente tensionadas pela questão agrária tratada por historiadores, antropólogos, cientistas políticos e sociólogos ^1^.

Esse estado de coisas se refletiu no perfil do corpo docente da instituição. O CPDA constituiu uma massa crítica de características únicas na história acadêmica do país, com alto nível de formação e diversificado, tanto em termos disciplinares, quanto de orientação doutrinária e temática. Economistas de formações variadas, desde neoclássicos como Affonso Celso Pastore, macroeconomista, e Antônio Carlos Nogueira, economista rural, até heterodoxos de espectro diversificado como Carlos Lessa, Yoshiaki Nakano, José Pereira Wilken Bicudo, Paulo Roberto Beskow, Nelson Giordano Delgado, Ana Célia Castro e Ivan de Otero Ribeiro, que estiveram ao lado da socióloga Miriam Limoeiro Cardoso; dos agrônomos Horácio Martins de Carvalho e Roberto Moreira, ambos com forte formação interdisciplinar, o primeiro especialista em planejamento com reconhecida capacidade em ciência política, o segundo com sólida formação em economia política; dos antropólogos Otávio Guilherme Velho e Margarida Maria Moura; e dos historiadores Maria Yedda Leite Linhares, Guillermo Palácios e Francisco Carlos Teixeira da Silva. Vale mencionar que esse corpo de professores e pesquisadores, pelas duplas, às vezes múltiplas afiliações institucionais, articulava o CPDA a diferentes centros de pensamento: Affonso Celso Pastores, por exemplo, era docente de economia da USP; Carlos Lessa, a sua vez, docente da CEPAL e da Universida Federal do Rio de Janeiro (UFRJ), e Yoshiaki Nakano, da Escola de Economia da FGV-SP; Otávio Guilherme Velho era já um proeminente professor de antropologia no Museu Nacional da UFRJ e Miriam Limoeiro Cardoso importante professora de sociologia da Pontifícia Universidade Católica do Rio de Janeiro (PUC-RJ); e por aí se segue.

Essa fase, que vai da sua criação até 1981, quando inicia um processo de transferência para a Universidade Federal Rural do Rio de Janeiro (UFRRJ), é conhecida como “CPDA do Horto”, em alusão ao fato do centro funcionar no Horto Florestal, no solar da antiga residência da monarquia no Rio de Janeiro, hoje Solar da Imperatriz ^1^. No CPDA do Horto, produziu-se, inicialmente, uma leitura crítica das abordagens dominantes da agricultura no Brasil, contribuindo com o amplo debate que se iniciava no âmbito universitário sobre as singularidades do desenvolvimento do país, em particular de sua dimensão agrária. Ao mesmo tempo, foram propostos caminhos de uma nova interpretação desse objeto. Constituíram-se eficientes catalisadores desse movimento: os programas de pesquisa mencionados.

O Programa de História da Agricultura era dirigido pela professora Maria Yedda Leite Linhares, catedrática da antiga Universidade do Brasil, atual UFRJ, aposentada compulsoriamente pela ditadura militar em 1969, professora associada em Paris-Vincennes e na Universidade de Toulouse-Le Mirail, na França, desde então, até voltar ao Brasil, em 1974, a Professora Maria Yedda desbravou a área de história da agricultura em nosso país, criando programas de pesquisa, como o já mencionado, na EIAP-FGV, e, após sua plena recuperação como professora do magistério público federal, outros na Universidade Federal Fluminense (UFF) e na UFRJ. Nesse seu profícuo percurso, orientou dezenas de dissertações e teses, incluindo a minha dissertação de mestrado.

Em torno do Programa de História da Agricultura, a professora Maria Yedda arregimentou um grande número de colaboradores, para além dos quadros do CPDA. Entre esses, Alfredo Wagner Berno de Almeida, Ciro Flamarion S. Cardoso, Maria Bárbara Levy, Eulália Maria Lahmeyer Lobo, Fernando Antônio Novais, Francisco José Calazans Falcon, João Pacheco de Oliveira Filho ^1^. Ademais, o Programa de História da Agricultura mantinha um evento semanal de discussões pelo qual desfilaram seus trabalhos nos salões da antiga monarquia no Horto Florestal do Rio de Janeiro, além dos professores e pesquisadores da casa, permanentes ou parciais, *scholars*, como Warren Dean, Octavio Ianni, José Manoel Cardoso de Melo, Manoel Maurício, Wilson Cano, Afrânio Garcia, Klaas Woortmann, Shepard Forman, Ismênia Martins, Ilmar Rohloff Mattos, Alice Canabrava e Maria Rita Garcia Loureiro. Sobre o clima vibrante daqueles dias, a professora Maria Yedda rememora em 2007: “*Jovens em busca de conhecimento, ideias ou reafirmação compunham um quadro permanente nos jardins do casarão. Lia-se avidamente Witold Kula, Georges Duby, Fernand Braudel, Karl Polanyi e Maurice Godelier e se discutiam os rumos do país nas vésperas do colapso da ditadura*” ^1^ (p. 166).

No Programa de História da Agricultura do CPDA do Horto, tornara-se premente o enfrentamento das restrições teóricas e metodológicas que caracterizavam a historiografia agrária no país, em particular dos limites impostos pelas interpretações dualistas da diversidade estrutural da realidade brasileira desenvolvidas nas últimas décadas.

Uma primeira linha de questionamento referia-se à abordagem que privilegiava a agricultura de exportação, obscurecendo tudo mais. Desde o trabalho seminal de Roberto C. Simonsen, em 1937 ^3^, que dividia o período colonial em ciclos dominados por um produto ou atividade - o “ciclo da indústria extrativa”, com destaque para o “pau-brasil”; o “ciclo do açúcar”; e o “ciclo da mineração” -, abordava-se a história econômica do país por sucessões desses ciclos, acrescendo-se, em seguida, o “ciclo do café”, o “ciclo da borracha” etc., tal perspectiva comprometia a visão histórica do país, seja referente aos seus fundamentos estruturais, seja em relação aos seus movimentos e evolução no tempo.

Em termos estruturais, a centralidade ou mesmo a exclusividade da economia exportadora (do pau-brasil, do açúcar, da mineração, do café) implicava na supressão da produção para o abastecimento interno (de arroz, milho, feijão, mandioca, cachaça) e, com essa ausência, obscurecia-se a presença efetiva de outras estruturas que não a plantation imediatamente responsável pelo ciclo. Uma ausência particularmente sentida era a das estruturas de pequenos produtores familiares, uma vez que se reuniam evidências de sua importância, com pesos variados, praticamente em todas as partes e momentos da história do país. Era o que informavam os trabalhos de Ciro Flamarion Cardoso, sobre a brecha camponesa no Brasil escravocrata; de Antônio Cândido, sobre os caipiras de São Paulo; de Margarida Maria Moura, sobre camponeses de Minas Gerais; de José de Souza Martins, sobre camponeses na fronteira do norte do Paraná, e de Otávio Guilherme Velho, João Pacheco de Oliveira, Alfredo Wagner Berno de Almeida, sobre camponeses na Amazônia ^4^ ^,^ ^5^ ^,^ ^6^ ^,^ ^7^.

Ademais, como orientação do movimento temporal da realidade social, a noção de ciclo carregava consigo um conteúdo teleológico pelo qual o produto destacado apresentava necessariamente um ciclo de vida completo - surge, evolui, decai e some. Todavia, esse não foi o caso de nenhum dos produtos importantes para a história econômica do país - a não ser a nobre exceção do pau-brasil.

Uma segunda linha de questões limitantes para a história da agricultura no Brasil, segundo a leitura do CPDA do Horto, falava a respeito do dualismo da agricultura no conjunto da sociedade brasileira - uma outra perspectiva da diversidade estrutural. Popularizadas inicialmente por Jacques Lambert e Roger Bastide, professores da Escola de Sociologia e Política da Universidade de São Paulo (USP), afirmava-se que os países de passado colonial apresentariam uma dualidade de estruturas, com um setor “aberto e moderno” e outro “fechado e arcaico”. No Brasil, portanto, oporiam-se o velho, representado pelo imobilismo do interior, do rural arcaico herdado do passado colonial, como sobrevivência de formas ultrapassadas; e o novo, representado pelo dinamismo do litoral moderno, com suas estruturas urbanas que se vinculavam à indústria, ao grande comércio e às formas avançadas de civilização. A explicação dessa realidade estaria na colonização que gerou o latifúndio de caráter feudal, rigidamente hierarquizado, constituindo unidades autossuficientes, cujo traço predominante seria a resistência à mudança ^8^ ^,^ ^9^.

No Instituto Superior de Estudos Brasileiros (ISEB), Hélio Jaguaribe, Nelson Werneck Sodré, Guerreiro Ramos, Ignácio Rangel, entre outros, produziram uma absorção marxista dessas teses, amplamente influenciada pela perspectiva oficial do stalinismo. Segundo essa visão, haveria uma sucessão rígida de modos de produção na trajetória histórica das sociedades ocidentais. Aplicada ao Brasil, a noção levou à percepção de que as estruturas econômicas e sociais do país se caracterizariam pela coexistência de dois modos de produção diferentes: o capitalista (moderno, manifesto na indústria e nos demais setores urbanos) e o feudal (arcaico, estabelecido na agricultura em formas latifundiárias e minifundiárias -, i. e. de grandes estabelecimentos baseados em relações não capitalistas de arrendamento, colonato ou parceria e de pequenos estabelecimentos camponeses). Na perspectiva que, nesse contexto, veio a se estabelecer como abordagem “nacional-desenvolvimentista”, o desenvolvimento do país dependeria do setor capitalista nacional (e da liderança de seus dirigentes, uma burguesia nacionalista), em oposição às empresas capitalistas estrangeiras (expressão do imperialismo) e aos feudos latifundiários. Desse processo de desenvolvimento, faria parte uma reforma agrária com dupla finalidade: garantir o abastecimento do mercado interno de alimentos a preços baixos, sendo necessário, para tanto, ampliar o campesinato e elevar sua produtividade; e, com a elevação do poder de compra dos camponeses, incrementar o mercado interno de bens industriais provindos da indústria nacional ^10^ ^,^ ^11^ ^,^ ^12^.

Na década que se seguiu ao golpe militar de 1964, os esquemas dualistas de interpretação do Brasil se mostraram frágeis, já que seus pressupostos teóricos - o caráter feudal das estruturas rurais e o nacionalismo como instrumento de condução da política contra o imperialismo - não foram comprovados empiricamente. Ademais, o modelo de desenvolvimento que se seguiu nos próximos anos, na indústria, baseou-se num arranjo que se mostrou virtuoso em promover o crescimento econômico entre empresas estrangeiras, empresas estatais e setores nacionais, assim como na agricultura e na modernização tecnológica do grande latifúndio com mudanças conexas nas relações de produção, o que mostrou a impropriedade do esquema analítico.

Duas linhas de confrontação crítica alcançaram inicialmente o dualismo. Uma apoiada no individualismo metodológico, instrumentado por abordagens neoclássicas do desenvolvimento do país e da agricultura, e outra estruturalista, de fundamentação marxista.

Na perspectiva neoclássica, formulada, por uma parte, na USP, sob a liderança de Antônio Delfim Netto, acompanhado de Affonso Celso Pastore, entre outros, e no Instituto Brasileiro de Economia (IBRE), FGV, por Rui Miller Paiva, o rural brasileiro estaria povoado por agentes, cujas decisões seriam compatíveis com a teoria econômica ortodoxa. Portanto, não haveria pertinência nos pressupostos dualistas de que existiriam obstáculos derivados de razões e modos de produção não capitalistas. Tanto seria assim, que o setor, em vez de se constituir em entrave, vinha, como um todo, reagindo às tensões que o crescimento urbano e industrial exercia sobre ele, de acordo com as expectativas teóricas das análises de mercado, já que vinha elevando a produtividade e, assim, produzindo alimentos a preços adequados e liberando mão de obra para a indústria. Ademais, o setor fornecia recursos para a formação de capital na indústria, além de constituir base de expansão do mercado consumidor. Por fim, o que explicaria as diferenças verificadas entre regiões e, nestas, entre atividades, seriam as diferentes dotações e produtividade dos fatores ^13^ ^,^ ^14^ ^,^ ^15^ ^,^ ^16^.

A frente marxista de críticas ao dualismo teve em dois autores sua vanguarda: Caio Prado Júnior ^17^, professor de economia política da USP, que, já no início dos anos quarenta, argumentava que as origens do Brasil eram capitalistas, uma vez que o empreendimento colonial português se inseria no contexto da expansão do capitalismo mercantil; em 1966, retoma as teses então defendidas e as atualiza para um contexto em que o capitalismo industrial avança no país sob a liderança de empresas estrangeiras, com desdobramentos incontornáveis na agricultura ^18^ ^,^ ^19^; e André Gunder Frank ^19^, em artigo publicado um pouco antes, em 1964, partindo também do caráter capitalista da colonização, argumenta que a industrialização do Brasil evolui de maneira subordinada ao imperialismo e com severa dependência tecnológica, sob a égide do capitalismo, que organiza o conjunto da sociedade, inclusive sua agricultura. Nessa perspectiva, é o funcionamento e o avanço desse sistema que produz, necessariamente, tanto o desenvolvimento, quanto o subdesenvolvimento.

Todas essas discussões reverberaram com força entre 1977 e 1981 no CPDA do Horto e constituíram base para reflexões em muitas direções. Primeiro, incomodava o fato de que, em ambas as frentes, com a rejeição do que era criticável no dualismo - uma diversidade fundamental entendida como realidades distintas lado a lado, estanques em relação uma à outra -, anulou-se toda diferença, na medida em que se estabeleceu para o diverso um estatuto de necessária transitoriedade: ao final, se as diversidades existiam, elas eram restos irrelevantes de formas anacrônicas que teimavam em continuar existindo, mas não por muito tempo. Não obstante as diferenças explanatórias (se a diversidade deriva da existência de agentes racionais e não-racionais ou de estruturas arcaicas em convívio com modernas), todos tendiam a ver o distinto como necessariamente (a lógica ou a história seriam implacáveis) e desejavelmente (o melhor devir pressupunha sua extinção) transitório. Tão rapidamente ocorreriam as superações, acreditavam todos, neoclássicos, marxistas, desenvolvimentistas, que deveríamos proceder como se eles, os distintos, já não importassem.

No CPDA do Horto, a crítica a esses entendimentos ensejou uma rediscussão do marxismo e suas noções fundamentais de modo de produção e formações econômico-sociais mesmo a partir da crítica ao estruturalismo, permitida pela utilização crescente do chamado Capítulo VI do *O Capita*l, manuscrito do próprio Marx, inédito até o início dos anos 1960, publicado no Brasil em 1978 ^20^. É importante assinalar que se fez essa releitura considerando as contribuições teóricas e metodológicas da Escola dos Annales, bases do importantíssimo curso de história da agricultura ministrado pela professora Maria Yedda Linhares. A partir daí, indagava-se sobre a capacidade do processo de desenvolvimento capitalista funcionalizar, criar e recriar formas não capitalistas de produção preexistentes ou emergentes e, assim, conter em seus modos de evolução diversidades estruturais de longa duração.

Sobressai daí ontologias que vão inquirir a agricultura brasileira como realidade multifacetada, a qual se deve procurar compreender a especificidade da formação econômico-social do país que, embora sob a hegemonia do capitalismo, articula diferentes relações e modos de produção. Mais detalhadamente, as grandes hipóteses seriam ^21^:

1) A agricultura brasileira se constituiu no contexto do desenvolvimento capitalista do país, caracterizado como dependente e periférico. As formas técnicas atrasadas e a multiplicidade de relações de trabalho no campo não se explicariam como sobrevivências coloniais, mas, sim, como formas de organização produtiva funcionalizadas pela própria racionalidade do capitalismo dependente e periférico;

2) Formas capitalistas de produção afirmavam-se crescentemente com base em diferentes formas de subordinação direta do trabalho, desde o assalariamento estável em certas circunstâncias e funções, até o trabalho volante temporário que tendia a se generalizar na grande cultura de exportação;

3) Formas camponesas de produção se encontravam em diversos formatos e contextos: produzindo alimentos como pequenos parceleiros nas áreas de colonização antiga mais deprimidas, ou como pequenos empresários rurais em áreas antigas mais dinâmicas, ou ainda como posseiros em áreas novas do país, na sua fronteira agrícola.

A Amazônia será problematizada nesta discussão, em fins dos anos setenta e início dos oitenta, como a mais recente área de fronteira agrícola do país. Sendo tal, ela será caracterizada, conforme demonstraram os trabalhos de Otávio Guilherme Velho e José de Souza Martins, como outras fronteiras agrícolas, por frentes camponesas de características e origens diversas em processos de colonização agrícola espontânea ou dirigida ^22^ ^,^ ^23^ ^,^ ^24^. Todavia, os trabalhos de Roberto de Araújo Santos, João Pacheco de Oliveira Filho e Armando Dias Mendes logo irão revelar que populações caboclas locais, grosseiramente desconsideradas na discussão brasileira, compunham estruturas precedentes a esse movimento de fronteira, trazendo características próprias para o processo ^25^ ^,^ ^26^ ^,^ ^27^.

A fronteira amazônica se mostrará distinta, também, nessa fase, como indicaram Octavio Ianni, Lúcio Flávio Pinto e José de Souza Martins, pela crescente participação de empreendimentos agropecuários capitalistas apoiados em mecanismos estatais de incentivos por concessões fundiárias, fiscais e creditícias ^28^ ^,^ ^29^ ^,^ ^30^ ^,^ ^31^. A componente capitalista da fronteira amazônica constituía fenômeno inédito na formação rural do país e não era prevista nos esquemas analíticos que acompanhavam a modernização do latifúndio, porque não resultava de processos de evolução de estruturas rurais preexistentes - não era iniciativa de fazendeiros locais, pecuaristas do Marajó, por exemplo, que se modernizavam, ou de fazendeiros de café de São Paulo, ou de cana de açúcar de Pernambuco, ou de cacau da Bahia, em processos de expansão ou mudança na longínqua Amazônia, mas, sim, empresas urbanas dos principais centros industriais e de serviços do país em processo estimulado de ruralização.

Essa forma de evolução da fronteira amazônica, em seus elementos decisivos, iniciara-se em fins dos anos sessenta, com a consolidação do conjunto de políticas que a ditadura militar impusera à região. Dessa “Operação Amazônia”, como foi designada na época, faziam parte instrumentos fundiários, fiscais e creditícios coordenados pela SUDAM, que favorecia as grandes empresas industriais e de serviços que viessem a atuar como empresas agropecuárias na Amazônia. Não obstante, em fins dos anos setenta, os resultados da estratégia não estavam claros, eis que nenhuma empresa ainda se consolidara. A observação das perspectivas futuras era toldada pela forte restrição ao acesso às informações das instituições envolvidas nessa operação, particularmente sensível, de um Estado particularmente obscuro em seu ethos autoritário. Apesar disso, duas dissertações de mestrado gestadas no CPDA do Horto se dedicaram a investigar o significado dessa inusitada presença do grande capital urbano na fronteira agrícola.

Maria das Graças Derengowski Fonseca ^32^, sob orientação de José Graziano da Silva, empreendeu uma corajosa e importante pesquisa de campo para tentar captar, em entrevistas diretas, o rumo das coisas. Seu trabalho findou por qualificar o movimento como aventureiro. Minha dissertação de mestrado, no contexto do Programa de História da Agricultura e sob a orientação da professora Maria Yedda Linhares, procurou acessar o significado do fenômeno por via indireta, resgatando a história do projeto Ford na Amazônia ^33^.

Pesquisar a experiência da Ford Motor Company na Amazônia dos anos 20 pareceu uma saída para a compreensão do que se passava. Ocorreu-nos, à minha orientadora, ao meu coorientador, professor Horácio Martins de Carvalho, e a mim, que essa impressionante experiência, àquela altura, muito pouco estudada, representava um protótipo das experiências agora em andamento na região sob a batuta da SUDAM: em essência, tanto antes, como agora, tratavam-se de empreendimentos capitalistas baseados em grandes *plantations* ou extensões de pasto lidando, a partir de uma lógica industrial estrita, com as peculiaridades naturais e sociais da região - estas, francamente incompreendidas, tanto no tempo da Ford, quanto nos tempos da modernização autoritária dos anos setenta, palco daquela nossa vivência acadêmica.

Realizada a pesquisa, constatou-se que nossas suposições estavam corretas: a experiência da Ford nos anos vinte e trinta estava pontilhada de situações equivalentes a fenômenos em andamento nos anos setenta na experiência da SUDAM, particularmente as tensões sociais em torno do trabalho de um campesinato extenso e multifacetado e as dificuldades tecnológicas que, conjuntamente, findaram por fazer fracassar o grande empreendimento. O aviso final que o trabalho oferecia aos protagonistas do projeto capitaneado pela SUDAM, meio século depois, era contundente: o vigente nos anos 1970 e 1980 poderia repetir o fracasso da Ford, possivelmente, pelas mesmas razões. Já com esses primeiros exercícios, estabeleceu-se em mim a disposição de testar a hipótese de que uma diversidade estrutural profunda, que abriga interações entre modos de produção, com eventos de negação, recriação e emergência históricos, portanto, sem lugar a teleologias, como apontava o CPDA do Horto, é uma característica de longa duração da formação rural brasileira que se manifestava com força no seu momento amazônico. Essa disposição organizou meu programa de trabalho desde então, iniciado pela tese de doutorado.

Danilo: Notamos que no centro de sua teoria está a categoria do “campesinato caboclo Amazônico”. Em que momento esta centralidade se estabelece? Em que trajetórias tecnoprodutivas é encontrada e como elas afetam a sobrevivência e qualidade de vida dessa população?

Chiquito: Descrevi até agora o *background* de minhas pesquisas que desembocaram no doutorado. Lá, no departamento de economia da Freie Universität Berlin, sob a orientação dos professores Manfred Nitsch e Elmer Altvater, de 1984 a 1988, investiguei a formação dos campesinatos amazônicos do período colonial até os anos 1980 do século XX. De volta ao Brasil, desenvolvi estudos da experiência da SUDAM, de ruralização na Amazônia de grandes empresas industriais, bancárias e comerciais, confrontando essa experiência, que de fato veio a se demonstrar fracassada, com o surgimento de novas e diferentes estruturas patronais ou capitalistas economicamente viáveis que, todavia, caracterizavam-se por serem tecnologicamente atrasadas; analisei também a evolução de diferentes campesinatos, que, ao contrário, ressurgiam de crises em diferentes fronteiras por inesperados movimentos de inovação tecnológica de grande criatividade, extensão e envergadura ^34^.

Parte desse esforço se constituiu no desenvolvimento de capacidade teórica e metodológica de tratar a diversidade estrutural como resultado da interação estrutura-razão-ação, aceitando, inicialmente, a sugestão de Antônio Carlos Kfouri Aidar & Roberto Mário Perosa Junior ^35^, de que há diferenciações relevantes nas razões decisórias dos agentes movidos por lucro, dado que podem se orientar por uma lógica mais mercantil, ou mais industrial, ou mais financeira, implicando, isso, nos resultados tecnológicos diferentes que víamos entre os tipos de empresas; logo me foi possível entender que essas distinções seriam tratáveis na perspectiva de John Maynard Keynes ^36^, de busca de eficiência marginal do capital por composição de portfólios variáveis, contingentes no tempo e no espaço. Por outro lado, desenvolvi, apoiado no trabalho seminal do economista russo Alexander Chayanov sobre a racionalidade econômica da economia doméstica, a categoria de eficiência reprodutiva, a partir da qual era possível explicar as dinâmicas e os resultados tecnológicos surpreendentes e variados que encontrávamos entre os camponeses ^37^.

Com isso, adquiri capacidade de visualizar a formação rural da Amazônia, por um lado, como resultado da evolução de formas camponesas, agregáveis em um modo camponês, em interação com formas patronais, e agregáveis em um modo patronal de organização da produção; por outro, como um processo no qual, referida a esses modos de produção observáveis em elevado nível de abstração, verificava-se uma grande diversidade de movimentos, situações estruturais e soluções tecnológicas concretas. Nossos estudos até 2005, sobre a formação agropecuária da Região Norte e das várzeas, são representativos do que com isso se logrou ^38^, ao mesmo tempo que expõem carências importantes: a de estabelecer sentidos sistêmicos para a variedade de fenômenos *ad hoc* que se descreviam concretamente e a de expor a coerência destes com suas grandes referências estruturais.

Quando fui para o Centro de Estudos Brasileiros do Faculdade de Santo Antônio da Universidade de Oxford, em 2007, levava esses problemas na bagagem. Visando superá-los, no tempo que lá estive, me aprofundei nos trabalhos de Paul David ^39^, um proeminente do lugar, sobre a centralidade das relações técnicas no crescimento econômico e dependência de trajetória na evolução dos procedimentos tecnológicos (sua mensagem central: a história importa); de Giovanni Dosi ^40^, sobre trajetórias tecnológicas como operações concretas referidas a padrões tecnológicos, ou paradigmáticos (os modos e as fontes de geração, seleção, difusão e apropriação de conhecimento tecnológico importam); e os de Brian Arthur ^41^, sobre concorrência de trajetórias tecnológicas como processo histórico, dependente do ambiente institucional e das diferenças posturais dos agentes (as razões e os modos de produção importam). A esse conjunto de resultados iluminadores, acresci a ideia de que, à historicidade do conhecimento e das instituições que eles destacam, atrelam-se territorialidades e, com elas, bases naturais necessárias (de modo que o lugar e a natureza importam) ^29^.

Com isso, desde então, passei a ter como hipótese de trabalho que as formas de utilização dos recursos naturais e institucionais, seus movimentos e conformações que concretamente víamos e descrevíamos, deveriam ser entendidos como pontos, momentos e situações de trajetórias tecnológicas, as quais poderiam eventualmente ser observadas mediante convergências no tempo e similaridades no espaço desses pontos ^42^. Para utilizarmos as bases de dados dos censos agropecuários e, assim, podermos ter delimitações dessas trajetórias em diferentes níveis de agregação, desenvolvemos um método que combina diferenciação e significação estrutural da produção rural em dois movimentos: no primeiro, segregam-se os modos de produção por características dos agentes e situação de suas estruturas e, no segundo, verifica-se como essas estruturas dessemelhantes abrigam convergências nas características da produção semanticamente associadas às trajetórias ^43^.

Os resultados, que já permitiam passos na interdisciplinaridade das ciências sociais, agora, com a exitosa experiência do projeto Trajetórias: *Serviços Ecossistêmicos como Serviços de Saúde: Trajetórias Competitivas para o Uso da Terra no Bioma Amazônia e sua Ligação com Doenças Transmitidas por Vetores*, promovido pelo Centro de Síntese em Biodiversidade e Serviços Ecossistêmicos, SinBiose do CNPq, parecem garantir saltos na transdisciplinaridade necessária ao tratamento complexo que o desenvolvimento da Amazônia, região crítica do planeta, merece.
